# Soluble Epoxide Hydrolase Inhibition Protected against Angiotensin II-induced Adventitial Remodeling

**DOI:** 10.1038/s41598-017-07512-1

**Published:** 2017-07-31

**Authors:** Chi Zhou, Jin Huang, Qing Li, Jiali Nie, Xizhen Xu, Dao Wen Wang

**Affiliations:** Division of Cardiology, Department of Internal Medicine, Tongji Hospital, Tongji Medical College of Huazhong University of Science and Technology, Hubei Key Laboratory of Genetics and Molecular Mechanisms of Cardiological Disorders, Wuhan, 430030 China

## Abstract

Epoxyeicosatrienoic acids (EETs), the metabolites of cytochrome P450 epoxygenases derived from arachidonic acid, exert important biological activities in maintaining cardiovascular homeostasis. Soluble epoxide hydrolase (sEH) hydrolyzes EETs to less biologically active dihydroxyeicosatrienoic acids. However, the effects of sEH inhibition on adventitial remodeling remain inconclusive. In this study, the adventitial remodeling model was established by continuous Ang II infusion for 2 weeks in C57BL/6 J mice, before which sEH inhibitor 1-trifluoromethoxyphenyl-3-(1-propionylpiperidin-4-yl) urea (TPPU) was administered by gavage. Adventitial remodeling was evaluated by histological analysis, western blot, immunofluorescent staining, calcium imaging, CCK-8 and transwell assay. Results showed that Ang II infusion significantly induced vessel wall thickening, collagen deposition, and overexpression of α-SMA and PCNA in aortic adventitia, respectively. Interestingly, these injuries were attenuated by TPPU administration. Additionally, TPPU pretreatment overtly prevented Ang II-induced primary adventitial fibroblasts activation, characterized by differentiation, proliferation, migration, and collagen synthesis via Ca^2+^-calcineurin/NFATc3 signaling pathway *in vitro*. In summary, our results suggest that inhibition of sEH could be considered as a novel therapeutic strategy to treat adventitial remodeling related disorders.

## Introduction

Aorta is composed of three tunicae: intima, media and adventitia. The roles of intima and media on vascular functions have been extensively studied, while the contribution of adventitia to vascular functions was recently recognized. Adventitial remodeling occurs during normal angiogenesis and under various pathological situations, including hypertension, atherosclerosis, aortic dissection, and restenosis after balloon angioplasty^1^. As the main cell types in adventitia, adventitial fibroblasts (AFs) can differentiate into myofibroblasts (MFs), proliferate, migrate, and secret extracellular matrix (ECM), which play a pivotal role in response to vascular injury^2^. Until now, the underlying mechanisms of adventitial remodeling are not fully understood, and there is no efficacious pharmacological therapy.

Angiotensin (Ang) II, a potent inducer that promotes phenotypic transition of fibroblasts to myofibroblasts, can increase intracellular Ca^2+^ concentration ([Ca^2+^]_i_) by interaction with AT1 receptor^3, 4^. Calcineurin (CaN) is a Ca^2+^-dependent serine/threonine protein phosphatase. The increased [Ca^2+^]_i_ binds to calmodulin (CaM) and regulates the enzymatic activity of calcineurin A subunit (CnA). Once activated, CaN combines and dephosphorylates cytoplasmic nuclear factor of activated T-cells (NFAT) transcription factors, which permits translocation of NFAT to nucleus and regulates pathological gene expression^5^. It has been reported that Calcineurin/NFAT signaling plays important roles in the pathogenesis of cardiovascular diseases. NFATc2 is a necessary mediator of calcineurin-dependent cardiac hypertrophy and heart failure^6^. Importantly, NFATc3 has specifically been implicated in vasculature development^7^. Bosc LV *et al*. proved that chronic hypoxia induced pulmonary hypertension and remodeling via calcineurin/NFATc3 signaling^8, 9^. Additionally, NFATc3 activation downregulates Ca^2+^-activated K^+^ channel in arterial smooth muscle and contributes to Ang II-induced hypertension^10^. Even though abundant studies have been reported, the precise mechanisms of calcineurin/NFAT signaling in adventitial remodeling process remain elusive.

It is well established that cytochrome P450 (CYP)/soluble epoxide hydrolase (sEH) system is widely expressed in human heart, vessel, liver, kidney, lung, and pancreas^11^. Epoxyeicosatrienoic acids (EETs) are synthesized from arachidonic acid (AA) by CYP epoxygenases, and sEH hydrolyzes EETs to less biologically active dihydroxyeicosatrienoic acids (DHETs). Accumulating evidences suggest that inhibition of sEH exerts important biological activities in various cardiovascular diseases. Previous study demonstrated that sEH is specifically upregulated by Ang II, and pharmacological inhibition of sEH prevents Ang II-induced cardiac hypertrophy^12^. Additionally, sEH inhibitor CDU was used as novel antihypertensive and antiatherosclerotic pharmaceutical by attenuating VSMCs proliferation^13^. Ephx2 gene deletion or sEH inhibition antagonize flow-induced neointimal formation and vascular remodeling^14^. These data suggest that CYP/sEH system is involved in the pathogenesis of vascular disorders. Recently, we found that cardiac-specific CYP2J2 overexpression protects against diabetic cardiomyopathy via inhibiting NFATc3 nuclear translocation^15^. Nonetheless, the underlying mechanisms of sEH inhibition on adventitial remodeling remain poorly understood.

In this study, we investigated the beneficial effects of sEH inhibition on Ang II-induced adventitial remodeling in C57BL/6 J mice. In addition, we used primary AFs treated with Ang II to explore the relevant mechanisms of sEH inhibition on Ca^2+^-calcineurin/NFAT signaling involved in adventitial remodeling process.

## Results

### TPPU administration increased vascular EETs levels in C57BL/6 J mice

**Figure 1:**
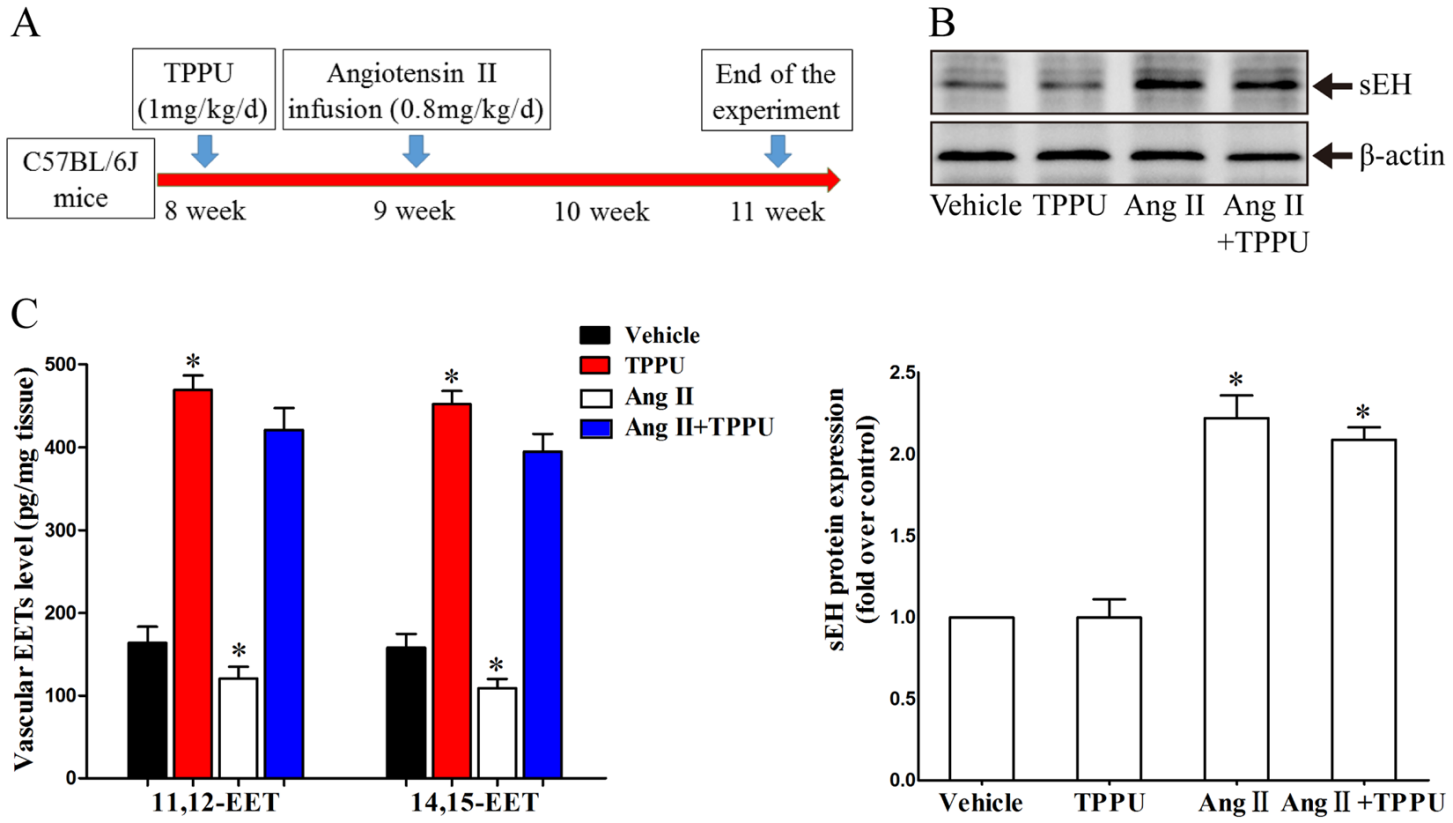
sEH was involved in adventitial remodeling process. (**A**) Schematic of animal model. (**B**) Representative immunoblots and quantitation of sEH protein expression in aortas of Ang II-infused mice. (**C**) Vascular 11,12-EET and 14,15-EET levels measured by LC/MS. Data were expressed as Mean ± SEM, n = 8 mice for each group. *p < 0.05 vs. Vehicle. Full-length blots/gels are presented in Supplementary Information.

To investigate the roles of sEH in adventitial remodeling process, we evaluated the level of sEH in continuous Ang II-infused mice model. As showed in Fig. 1B, Ang II infusion significantly increased sEH protein expression in aortic vessels, while sEH pharmacological inhibitor TPPU did not alter Ang II-induced elevation of sEH expression. Additionally, TPPU administration did not affect the aortic sEH expression in C57BL/6 J mice. These data were in consistent with previous study observed in Ang II-induced hypertrophic hearts^12^. sEH can hydrolyze EETs to less biologically active DHETs. As expected, sEH pharmacological inhibitor TPPU administration overtly increased vascular 11,12-EET and 14,15-EET levels, determined by LC/MS in mice (Fig. 1C). Meanwhile, Ang II infusion slightly decreased vascular EETs levels. Taken together, these results indicated that TPPU administration inhibited sEH enzyme activity and increased vascular EETs levels in C57BL/6 J mice.

### TPPU administration attenuated Ang II-induced adventitial remodeling in C57BL/6 J mice

**Figure 2:**
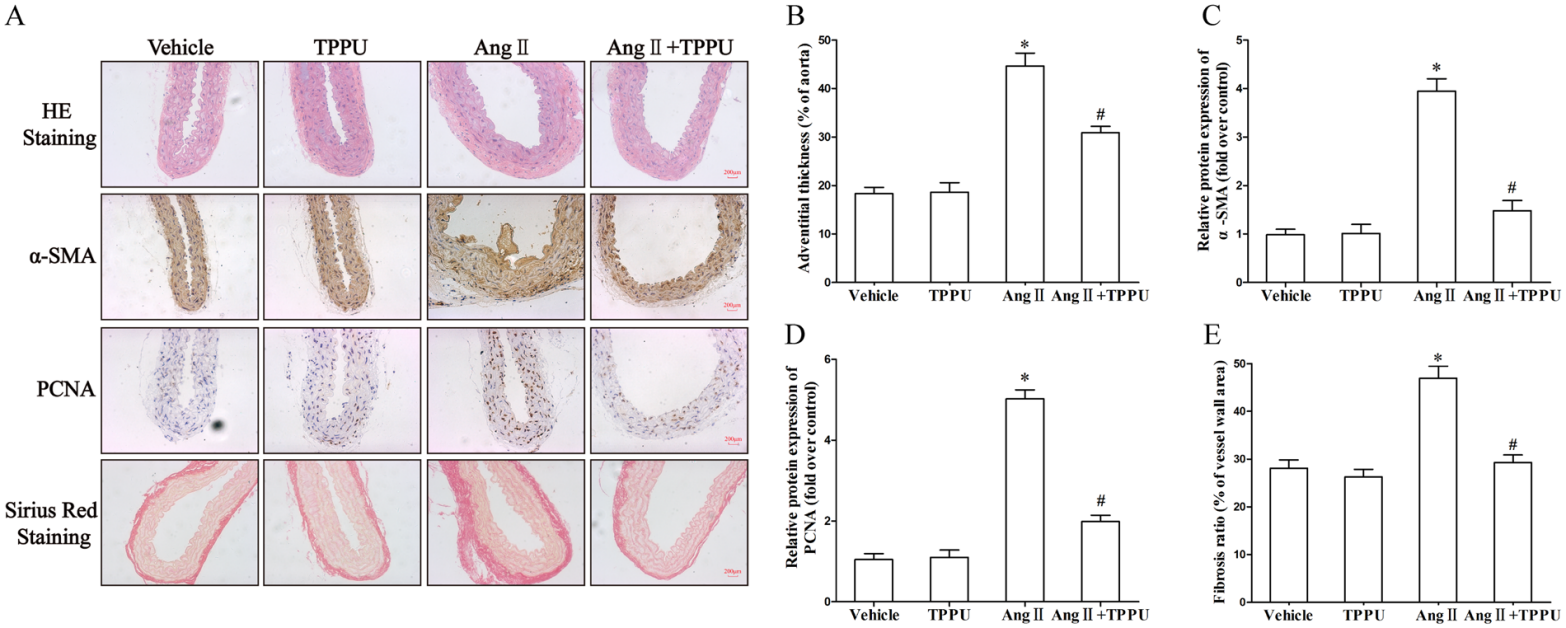
TPPU administration attenuated Ang II-induced adventitial remodeling in C57BL/6 J mice. (**A**) Vessel sections were performed with hematoxylin and eosin staining, sirius red staining, and immunohistochemical staining of α-SMA and PCNA. (**B**) Quantitative analysis of adventitial thickness. (**C**) Quantitative analysis of adventitial α-SMA expression. (**D**) Quantitative analysis of adventitial PCNA expression. (**E**) Quantitative analysis of adventitial fibrosis. Data were expressed as Mean ± SEM, n = 8 mice for each group. *p < 0.05 vs. Vehicle, ^#^p < 0.05 vs. Ang II.

Adventitial remodeling is characterized by adventitia thickening, phenotypic transformation of AFs to MFs, and extracellular matrix deposition^1^. In this study, we found that Ang II infusion led to obvious increases in both α-SMA and PCNA protein expression, determined by immunohistochemical staining in adventitia. However, TPPU treatment successfully prevented these effects. Further, the adventitial thickness in histological sections of mice from Ang II infusion alone was greater than those of vehicle, which confirmed the adventitial remodeling process, but was alleviated after TPPU treatment. In addition, the extent of adventitial collagen deposition was represent as fibrosis ratio (%), which is the proportion of vascular collagen area (Sirius red stain positive) to vessel wall area. These data defined the composition of the thickened vessel wall. Interestingly, Ang II infusion significantly increased collagen accumulation in adventitia, while TPPU administration remarkably reduced this effect. These results indicated that TPPU treatment inhibited Ang II-induced adventitial remodeling (Fig. 2).

**Figure 3:**
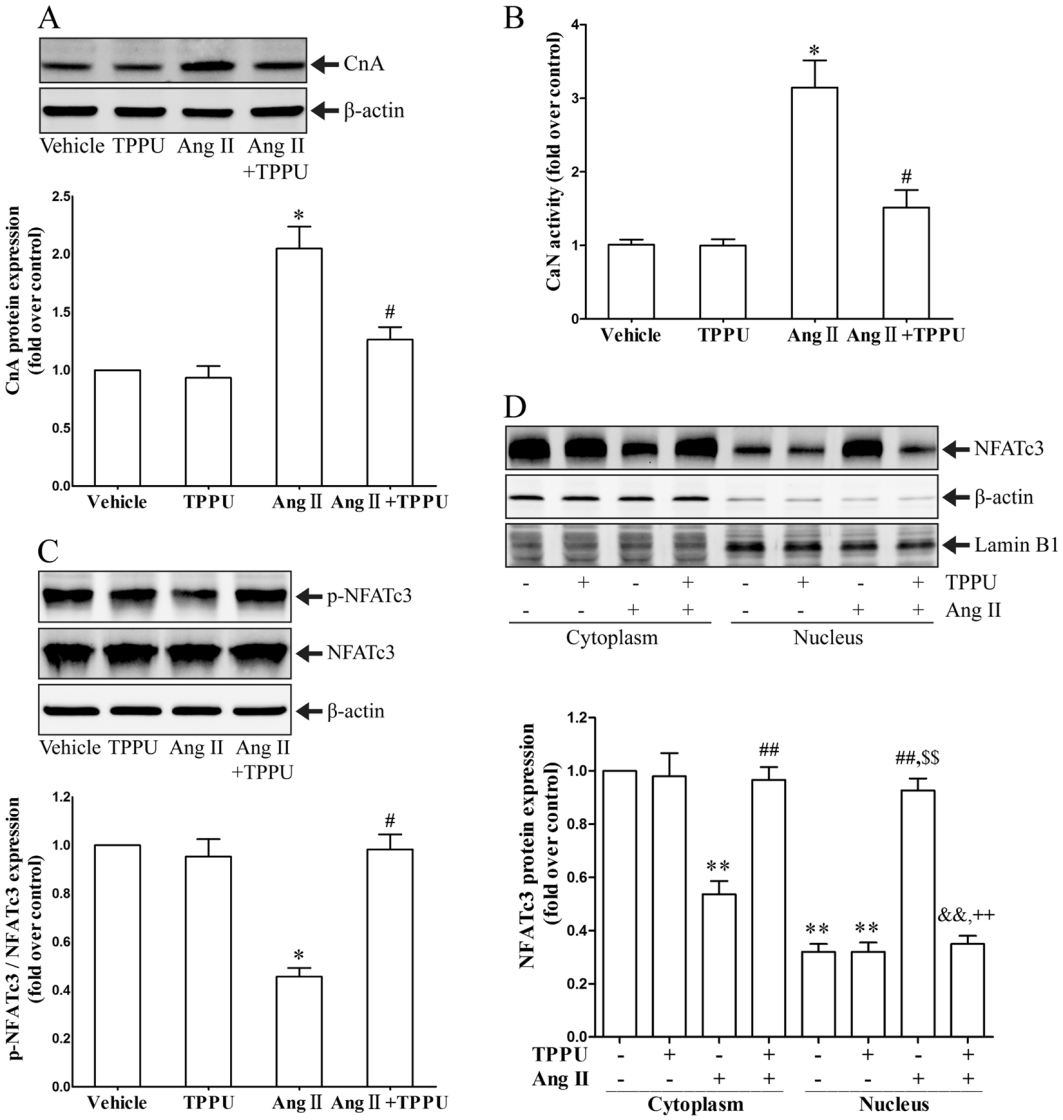
TPPU administration regulated Calcineurin/NFATc3 signaling in adventitial remodeling mice. (**A**) Representative immunoblots and quantitation of CnA protein expression in aortas. (**B**) Measurement of CaN phosphatase activity in aortas. (**C**) Representative immunoblots and quantitation of p-NFATc3/NFATc3 expression in aortas. (**D**) Representative immunoblots and quantitation of NFATc3 nuclear translocation in aortas. Data were expressed as Mean ± SEM, n = 8 mice for each group. *p < 0.05 vs. Vehicle, ^#^p < 0.05 vs. Ang II, **p < 0.05 vs. Vehicle (Cytoplasm), ^##^p < 0.05 vs. Ang II (Cytoplasm), ^&&^p < 0.05 vs. Ang II + TPPU (Cytoplasm), ^$$^p < 0.05 vs. Vehicle (Nucleus), ^++^p < 0.05 vs. Ang II (Nucleus).

To further explore the underlying mechanisms of sEH inhibition on adventitial remodeling, we examined the activation of calcineurin/NFATc3 signaling in mice aortas. As shown in Fig. 3A and B, Ang II infusion increased calcineurin protein expression and enzyme activity in aortic vessels, while TPPU administration alleviated these effects. Additionally, NFATc3 was primarily existed in the cytoplasm of vessel cells without stimulation. Ang II infusion significantly decreased cytoplasmic NFATc3 level, but increased nuclear NFATc3 level. Interestingly, TPPU treatment prevented Ang II-induced NFATc3 dephosphorylation and nuclear translocation in mice aortas (Fig. 3C and D). In summary, these findings revealed the beneficial roles of TPPU administration against Ang II-induced calcineurin/NATc3 activating in adventitial remodeling.

### TPPU significantly prevented the activation of primary AFs induced by Ang II

**Figure 4:**
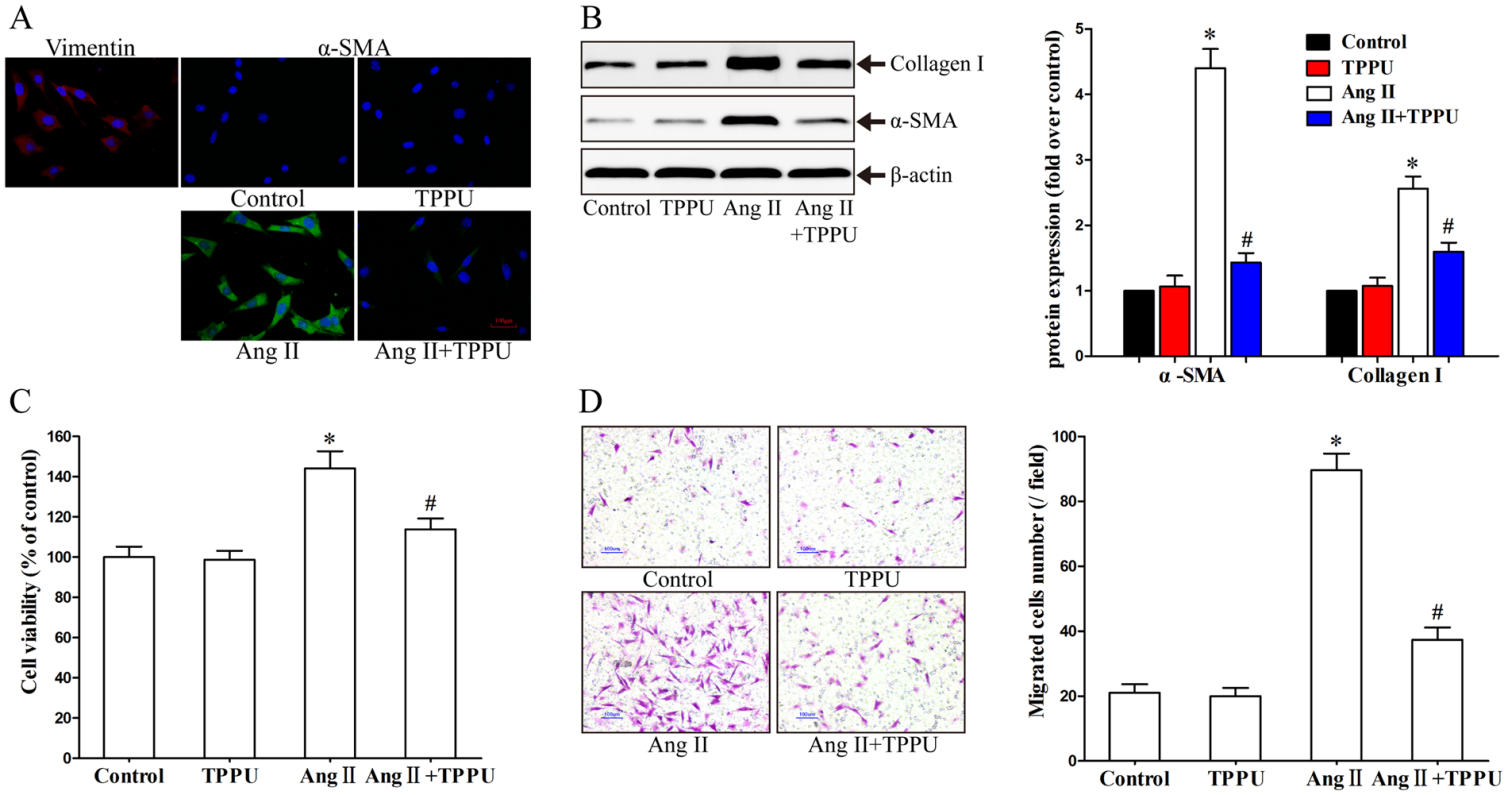
Effects of TPPU pretreatment on Ang II-induced AFs activation. (**A**) Immunofluorescent staining of Vimentin and α-SMA in AFs (Red: Cy3, Green: FITC, blue: DAPI). (**B**) Representative immunoblots and quantitation of α-SMA and Collagen I protein expression in Ang II-stimulated AFs pretreated with or without TPPU. (**C**) Measurement of cell proliferation by CCK-8 assay in Ang II-stimulated AFs pretreated with or without TPPU. (**D**) Measurement of cell migration by transwell assay in Ang II-stimulated AFs pretreated with or without TPPU. Data were expressed as mean ± SEM, n = 4 for each group, *p < 0.05 vs. Control, ^#^p < 0.05 vs. Ang II.

Firstly, immunofluorescent data revealed that the cultured cells were stained negative for α-SMA (VSMCs marker), but positive for Vimentin (AFs marker), which suggested the primary AFs were successfully isolated with a high purity (Fig. 4A). Ang II is a potent inducer that promotes phenotypic transition of AFs into MFs^3^. As demonstrated in Fig. 4A, the basal α-SMA level in AFs was very low, and Ang II remarkably increased the α-SMA expression, while TPPU pretreatment inhibited this transition. These results were further confirmed by western blot that TPPU significantly reduced the protein expression of α-SMA in Ang II-treated primary adventitial fibroblasts (Fig. 4B). Collagen I represents a major structural protein of the normal and diseased arterial wall^16^. We noticed that TPPU addition obviously prevented Ang II-induced collagen I synthesis in AFs (Fig. 4B). Phenotypic switching of AFs to MFs were accompanied by accelerated proliferation and migration^2^. Compared with control group, the absorbance of AFs in Ang II group increased by 44.01%. Interestingly, a significant anti-proliferative effect was observed after TPPU addition (Fig. 4C). Additionally, TPPU pretreatment substantially inhibited the migratory capacity of AFs induced by Ang II, as indicated by a marked decrease in the number of migrated cells (Fig. 4D). In aggregate, these data suggested that sEH inhibition by TPPU prevent Ang II-induced AFs differentiation, proliferation, migration and collagen synthesis.

### TPPU inhibited Ca^2+^-Calcineurin/NFATc3 signaling activation in Ang II-stimulated AFs

**Figure 5:**
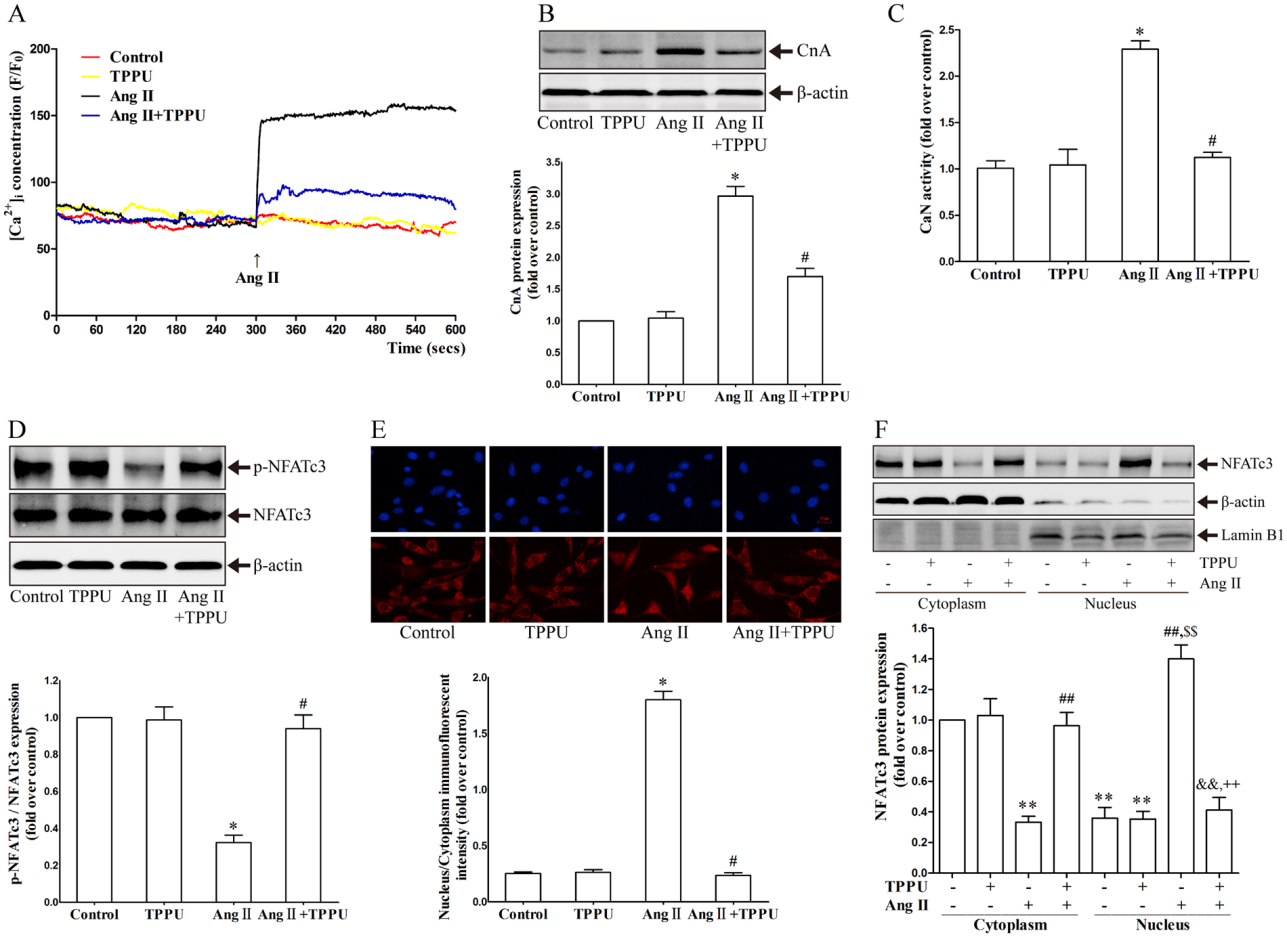
TPPU inhibited Ang II-activated Ca^2+^-calcineurin/NFATc3 signaling in AFs. (**A**) TPPU prevented Ang II-increased intracellular Ca^2+^ concentration in AFs. (**B**) Representative immunoblots and quantitation of CnA protein expression in Ang II-stimulated AFs pretreated with or without TPPU. (**C**) Measurement of CaN phosphatase activity in Ang II-stimulated AFs pretreated with or without TPPU. (**D**) Representative immunoblots and quantitation of p-NFATc3/NFATc3 expression in Ang II-stimulated AFs pretreated with or without TPPU. (**E**) Immunofluorescent staining of NFATc3 in Ang II-stimulated AFs pretreated with or without TPPU (Red: Cy3, blue: DAPI). (**F**) Representative immunoblots and quantitation of NFATc3 nuclear translocation in Ang II-stimulated AFs pretreated with or without TPPU. Data were expressed as mean ± SEM, n = 4 for each group, *p < 0.05 vs. Control, ^#^p < 0.05 vs. Ang II, **p < 0.05 vs. Control (Cytoplasm), ^##^p < 0.05 vs. Ang II (Cytoplasm), ^&&^p < 0.05 vs. Ang II + TPPU (Cytoplasm), ^$$^p < 0.05 vs. Control (Nucleus), ^++^p < 0.05 vs. Ang II (Nucleus).

It has been confirmed that calcineurin/NFAT signaling plays an important role in cardiac fibrosis^17^. The persistent increase in [Ca^2+^]_i_ is required to activate calcineurin/NFAT signaling and maintain cells function in pathological cardiovascular stress^18^. Our data proved that Ang II stimulation induced a rapid increase in [Ca^2+^]_i_ by 2-fold in AFs. In contrast, the elevation of [Ca^2+^]_i_ was significantly reduced by TPPU in comparison with Ang II-treated group (Fig. 5A). These results raised the possibility that TPPU might affect calcineurin/NFAT signaling. To test this hypothesis, we explored the activation of calcineurin/NFATc3 signaling in AFs. Interestingly, the protein expression and enzyme activity of calcineurin dramatically increased in response to Ang II, while TPPU addition blunted these activations (Fig. 5B and C). Further, NFATc3 was significantly dephosphorylated by Ang II, while this activation was prevented after TPPU treatment (Fig. 5D). Immunofluorescent data revealed that NFATc3 was primarily localized in the cytoplasm of AFs. Ang II stimulation evoked the NFATc3 nuclear accumulation, while TPPU addition significantly inhibited NFAT3 nuclear translocation compared with that in Ang II group (Fig. 5E). Similar results were observed by western blot that TPPU treatment prevented Ang II-induced NFATc3 nuclear translocation in AFs (Fig. 5F). These data consolidated the pivotal role of TPPU in the activation of AFs via Ca^2+^-Calcineurin/NFATc3 signaling pathway.

## Discussion

**Figure 6:**
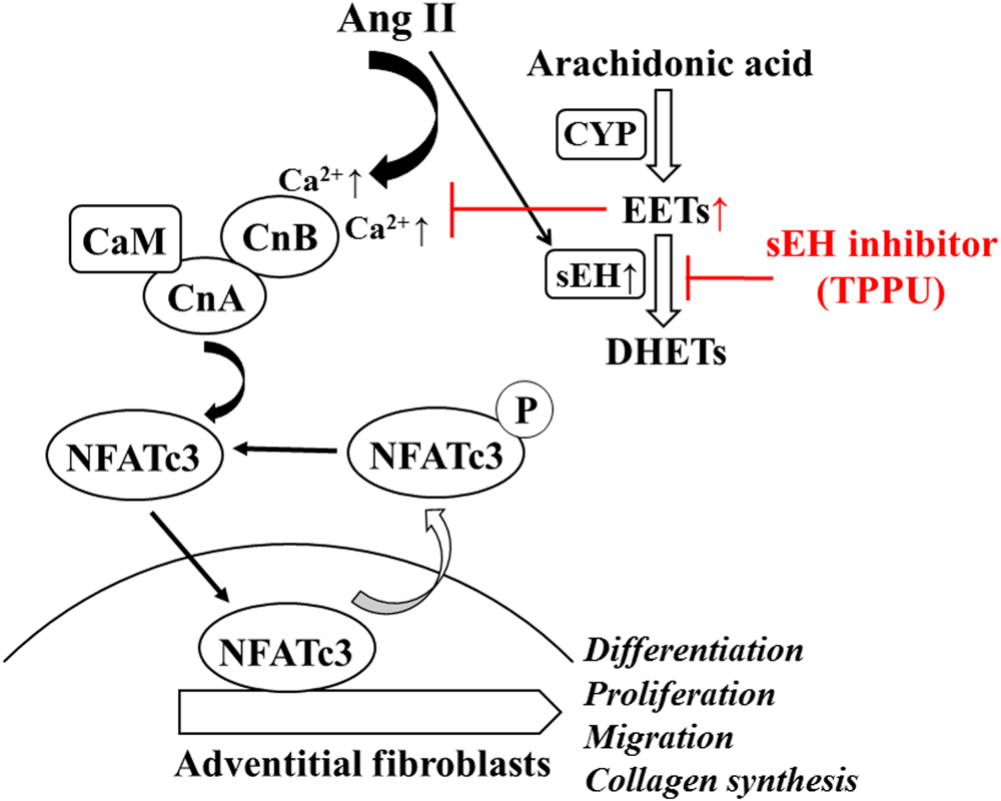
Schematic of the potential mechanisms of sEH inhibition on Ang II-induced adventitial remodeling.

In this study, the effects of sEH inhibition on Ang II-induced adventitial remodeling were investigated *in vivo* and *in vitro*. The novel findings indicated that sEH inhibition by TPPU significantly prevented Ang II-induced adventitial remodeling, characterized by adventitial fibroblasts differentiation, proliferation, migration, and collagen synthesis. Importantly, we provided evidences that TPPU exerted protective effects via inhibiting Ca^2+^-calcineurin/NFATc3 signaling pathway as illustrated in Fig. 6. These results suggested that sEH inhibition could be considered as a novel approach to treat adventitial remodeling related disorders.

Adventitia, as the outermost part of blood vessel, is an essential supportive layer of vessel, however, increasing evidences suggest that it also plays important roles in maintaining the homeostasis of vessel wall. Adventitia is activated and responds to vascular injury by an “outside-in” mechanism^19^. Adventitial fibroblasts, as the major cell type of adventitia, response to local environmental changes by transforming to myofibroblasts^20^. Our previous study have showed that CYP2J2 overexpression prevented adventitial remodeling, but the intrinsic mechanisms are not completely illustrated^16^. Soluble epoxide hydrolase converts cardiovascular protective EETs to less active DHETs. In this study, we excitingly observed that Ang II infusion increased sEH protein expression in mice aortas, which suggested that inhibition of sEH acitivity may be a potential therapeutic target of vascular diseases. TPPU is one of the most recently synthesized, stable, potent sEH inhibitors. As expected, TPPU treatment inhibited sEH enzyme activity, rather than impacting sEH protein expression, which increased vascular EETs levels in mice. The increased 11,12- and 14,15-EETs are vary with the placement of the epoxide group, which exerted pivotal roles in vascular homeostasis^21^. In this study, we excitingly observed that TPPU administration significantly prevent Ang II-induced adventitial thickening, phenotypic transition, AFs proliferation and ECM deposition in aortic adventitia. Besides, Ang II stimulated primary AFs activation *in vitro*, as evidence by differentiation, proliferation, migration and collagen synthesis, while sEH inhibition by TPPU reversed these effects. Our data were in consistent with previous studies that TPPU attenuated bleomycin-induced pulmonary fibrosis^22^ and cardiac fibrosis post-myocardial infarction^23^. Taken together, these observations indicated that sEH inhibition exerted beneficial effects against Ang II-induced adventitial remodeling.

We further investigated the underlying mechanisms involved in the protective effects of TPPU. It is known that Ang II could trigger Ca^2+^ influx, which mediated cardiac hypertrophy, cardiac fibroblasts differentiation, ECM deposition, and fibrogenic cytokines secretion through calcium signaling^24, 25^. Meanwhile, calcineurin/NFATc3 signaling is involved in chronic hypoxia-induced pulmonary vascular remodeling^8^. We recently reported that CYP2J2 overexpression relieved calcium overload and NFATc3 nuclear accumulation in diabetic cardiomyopathy^15^. However, whether sEH inhibition could suppress Ca^2+^-calcineurin/NFATc3 signaling in adventitial remodeling process remains inconclusive. In our study, Ang II stimulation caused intrcellular calcium overload in AFs, which leads to the activation of calcineurin phosphatase, NFATc3 dephosphorylation and nuclear translocation. The induction of NFATc3 activation was required for Ang II-induced AFs phenotypic transition and adventitial remodeling. Interestingly, sEH inhibition by TPPU overtly reversed Ang II-stimulated activation of Ca^2+^-calcineurin/NFATc3 signaling pathway. Our results revealed that the adventitial protective effects of sEH inhibition was likely due to its regulation on Ca^2+^-calcineurin/NFATc3 signaling.

In conclusion, the present study provided convincing evidences for the first time that sEH inhibition remarkably attenuated Ang II-induced adventitial remodeling via inhibiting Ca^2+^-calcineurin/NFATc3 signaling pathway in adventitial fibroblasts. These results suggested that sEH inhibition may serve as a novel therapeutic target for adventitial remodeling related disorders.

## Materials and Methods

### Adventitial remodeling animal model

All animal care and experimental procedures were approved by the Experimental Animal Research Committee of Tongji Medical College, Huazhong University of Science & Technology, and in strict accordance with the recommendations in the Guide for the Care and Use of Laboratory Animals of the NIH. 8 week-old C57BL/6 J male mice were provided by SLAC Laboratory Animal Co, Ltd (Shanghai, China), and housed in a temperature-controlled room under 12 h/12h-light/dark. Adventitial remodeling model was induced by a 14 days continuous Ang II (800 μg/kg/d) infusion by subcutaneous osmotic pumps (Alzet, Model 1002, USA) implantation (Fig. 1A)^16^. Mice were randomly divided into 4 groups: Vehicle group, TPPU group, Ang II group, and Ang II + TPPU group. TPPU (1 mg/kg/d) dissolved in 20% (v/v) PEG400 was administered by gavage 1 week before the induction. Each group included 8 mice.

### Evaluation of vascular EETs levels

Vascular EETs levels were analyzed using LC/MS as previously described^16^.

### Histological analysis

Mice thoracic aorta samples were separated and fixed in 4% paraformaldehyde, then dehydrated in graded ethanol series, embedded in paraffin, and sliced at 5 μm. Sections were performed with hematoxylin and eosin staining, sirius red staining, and immunohistochemical analysis of α-SMA and PCNA according to the manufacturer’s instructions.

### Isolation of primary adventitial fibroblasts

Adventitial fibroblasts were isolated from thoracic aortas of 12 week-old male C57BL/6 J mice as described previously with some modification^26^. Briefly, mice were anesthetized and then killed by vertebral dislocation, the thoracic aortas were removed and cleaned under sterilization. The medium was separated gently from the adventitia. The adventitia was minced carefully into small pieces (1mm^3^) in FBS, placed on 0.1% gelatin-coated dishes and cultured with 10% FBS high glucose DMEM containing 100 U/mL penicillin and streptomycin. The explants were incubated in a humidified incubator at 37 °C with 5% CO_2_. AFs grew out from tissues 3 days later, and reached confluence after 7–10 days. Experiments were performed on passages 3–6. Primary AFs were incubated with Ang II (10^−7^ M) for 24 h in the presence or absence of TPPU (10 μM).

### Immunofluorescent staining

AFs were fixed in 4% paraformaldehyde for 30 min, then permeated by 0.5% Triton X-100 for 30 min and blocked with goat serum for 2 h at room temperature. The primary antibodies against Vimentin, α-SMA, and NFATc3 at concentration of 1:100 were incubated at 4 °C overnight, followed by incubation with FITC or Cy3 labelled secondary antibody (1:100) for 2 h at room temperature avoid from light. Nuclei was stained with DAPI. Samples were observed by fluorescence microscope.

### Western blot analysis

Cells and tissue samples were extracted and homogenized, and nuclear protein was extracted using Boster Kit. The protein concentration was measured using BCA Kit (Boster, China). Totally 40 μg protein was subjected to SDS-PAGE gel for separation, then transferred into PVDF membrane. After blocking with 5% non-fat milk for 2 h, the membranes were incubated with primary antibodies against sEH, α-SMA, Collagen I, CnA, p-NFATc3, NFATc3, Lamin B1, β-actin overnight at 4 °C. After washing by TBST, the membranes were incubated with horseradish peroxidase-conjugated antibody for 2 h at room temperature. Bands were visualized using ECL and quantified with Gel Pro analysis software. β-actin and Lamin B1 were used as reference for cytoplasmic and nuclear protein, respectively.

### AFs proliferation assay

CCK-8 (Dojindo, Japan) assay was used to detect cell proliferation as described previously^16^. The relative cell viability was calculated as (OD_450treated_ − OD_450control_)/(OD_450untreated_ − OD_450control_) × 100%. Each experiment groups included 5 duplicate wells and repeated 4 times.

### AFs migration assay

Cell migration was evaluated by transwell cell culture chambers with 8.0 μm pores size polycarbonate membrane as described before (Corning, USA)^16^. The number of migrated AFs were quantified by counting 5 random fields (×100) for each membrane.

### Measurement of intracellular Ca^2+^ concentration

AFs were incubated with 5 μM Fluo-4-AM in Tyrode’s solution (mM: 136 NaCl, 4 KCl, 0.33 NaH_2_PO_4_, 4 NaHCO_3_, 2 CaCl_2_, 1.6 MgCl_2_, 10 HEPES, and 10 glucose, PH 7.4) at 37 °C for 30 minutes, followed by washing with Tyrode’s solution for 5 minutes before measurement^27^. The intracellular Ca^2+^ concentration was expressed as F/F_0_ (F_0_ represented the baseline fluorescence intensity).

### CaN phosphatase activity

CaN phosphatase activity in vessel tissues and AFs were detected using Calcineurin assay kit (Nanjing Jiancheng, China) according to the manufacturer’s instructions.

### Statistical analysis

All the experiment data were expressed as means ± SEM, and analyzed with SPSS 17.0 statistical software. Comparisons among groups were performed by one-way ANOVA with post hoc analysis using the Student-Newman-Keuls method. p < 0.05 was considered as statistically significant.

## Electronic supplementary material

Supplementary Information
