# Supplementary material for: Soluble Epoxide Hydrolase Inhibition Protected against Angiotensin II-induced Adventitial Remodeling
Source: Sci Rep. 2017 Jul 31;7:6926. doi: 10.1038/s41598-017-07512-1 (PMC5537243; doi:10.1038/s41598-017-07512-1)
Supplement: Supplementary file 1 — Supplementary Information [file 41598_2017_7512_MOESM1_ESM.pdf]

Soluble Epoxide Hydrolase Inhibition Protected against Angiotensin II-induced Adventitial Remodeling

Chi Zhou, Jin Huang, Qing Li, Jiali Nie, Xizhen Xu, Dao Wen Wang

Division of Cardiology, Department of Internal Medicine, Tongji Hospital, Tongji Medical College of Huazhong University of Science and Technology, Hubei Key Laboratory of Genetics and Molecular Mechanisms of Cardiological Disorders, Wuhan 430030, China;

Full-length gels and blots

The cropped gels/blots were processed without changing brightness and contrast of the images by Adobe Photoshop and Illustrator software.

Figure 1B

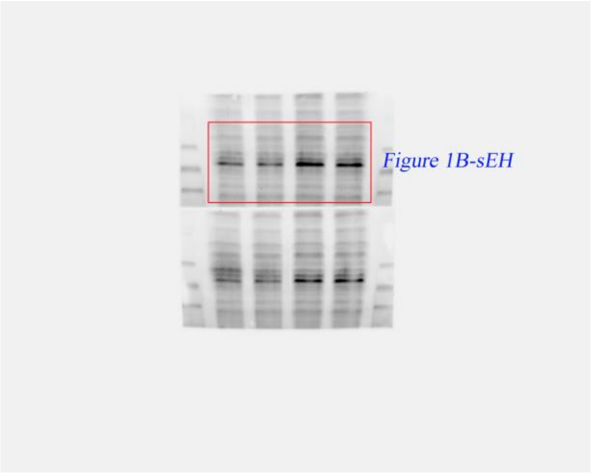

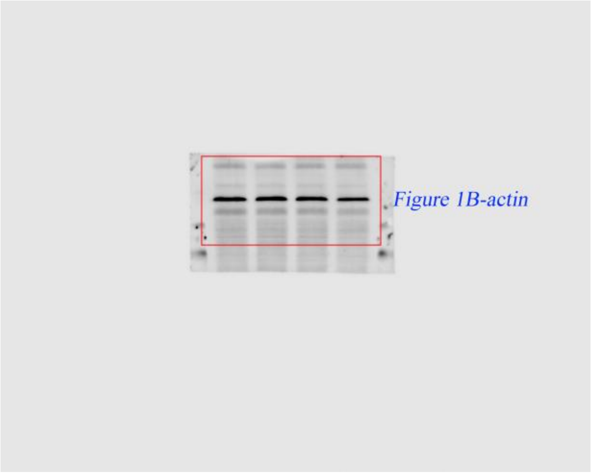

Figure 3A

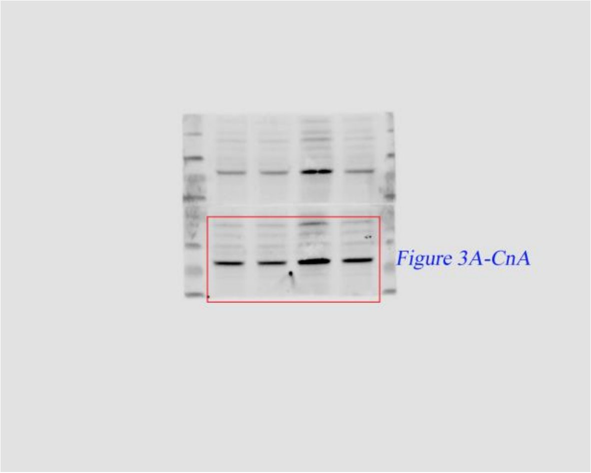

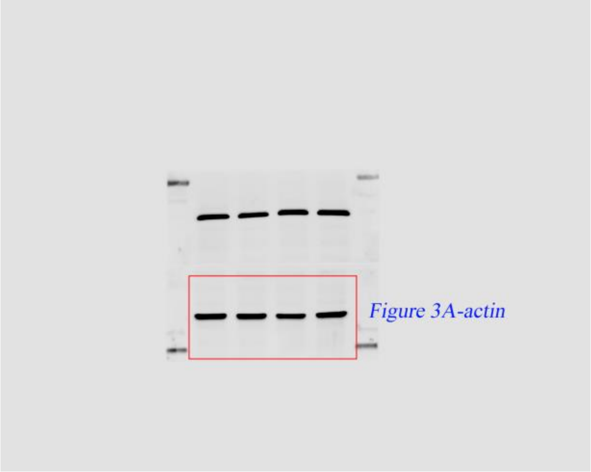

Figure 3C

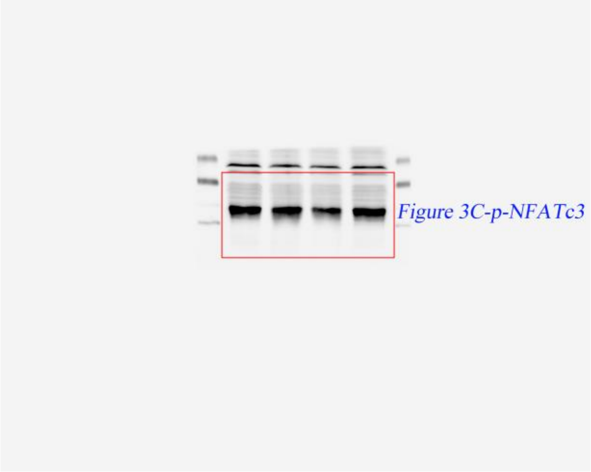

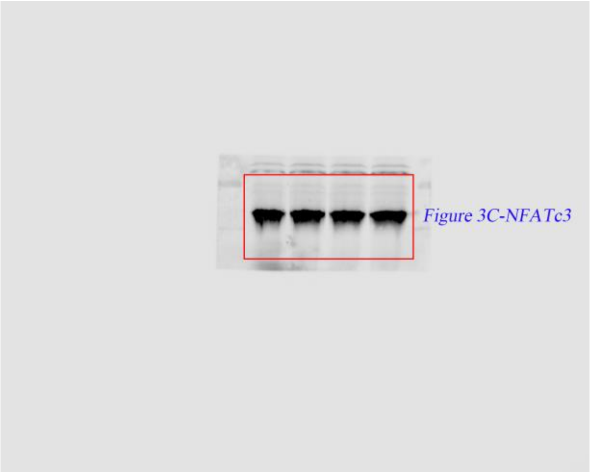

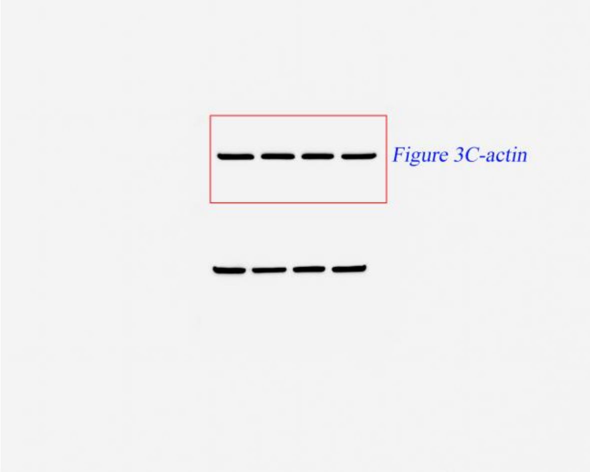

Figure 3D

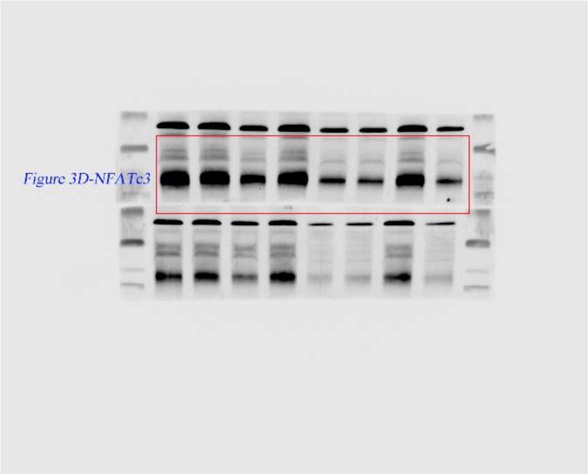

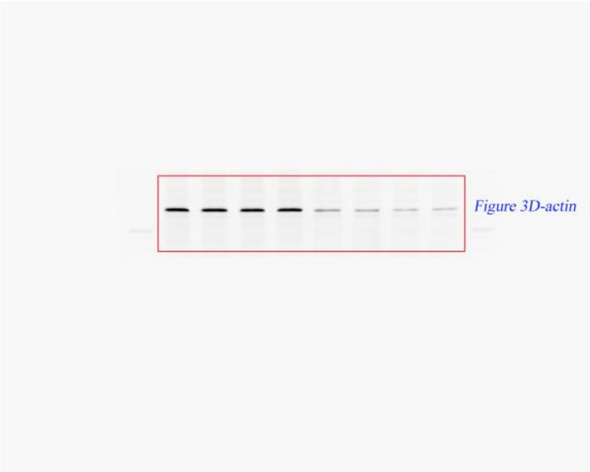

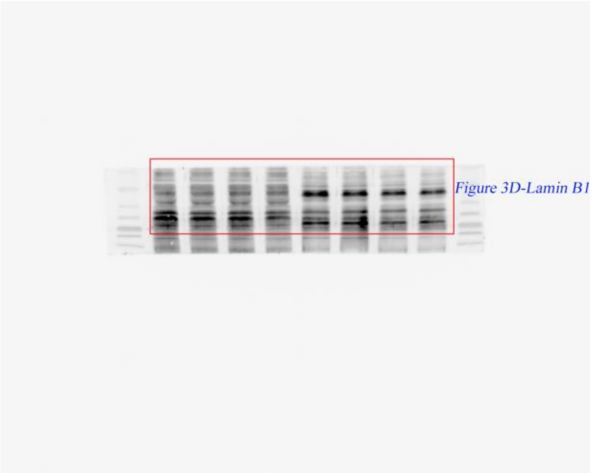

Figure 4B

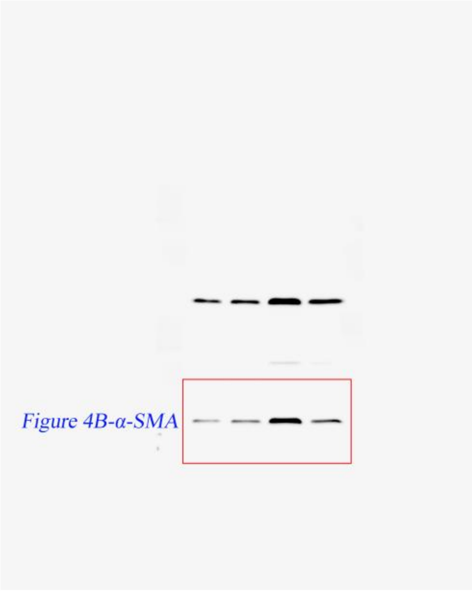

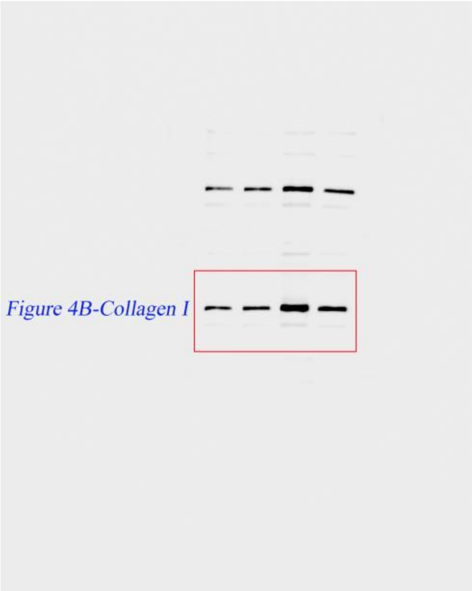

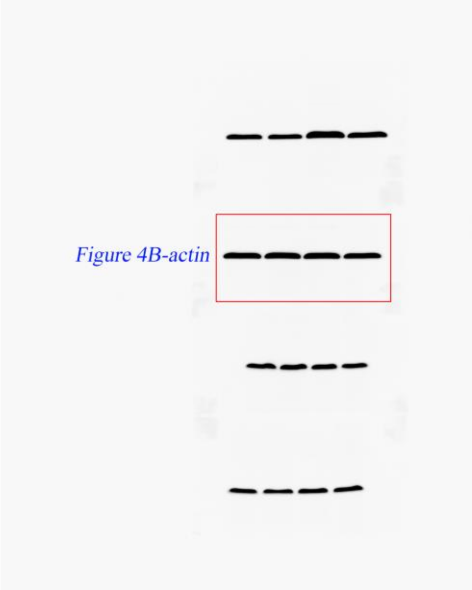

Figure 5B

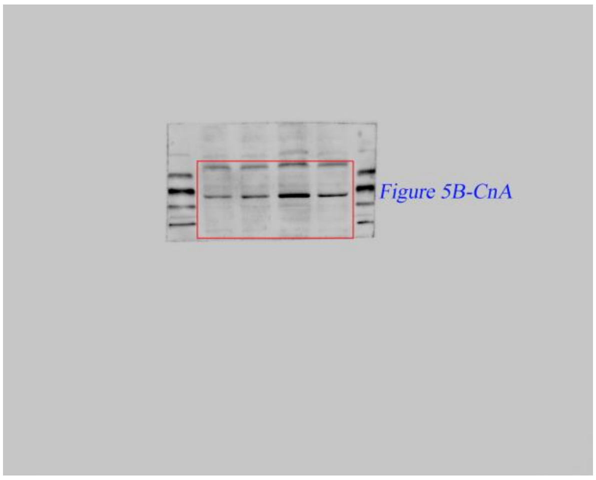

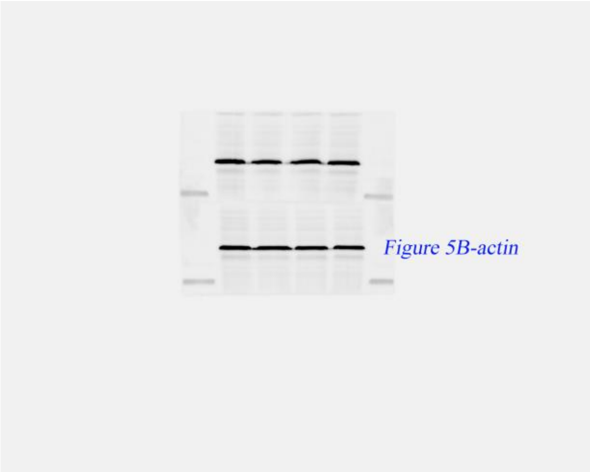

Figure 5D

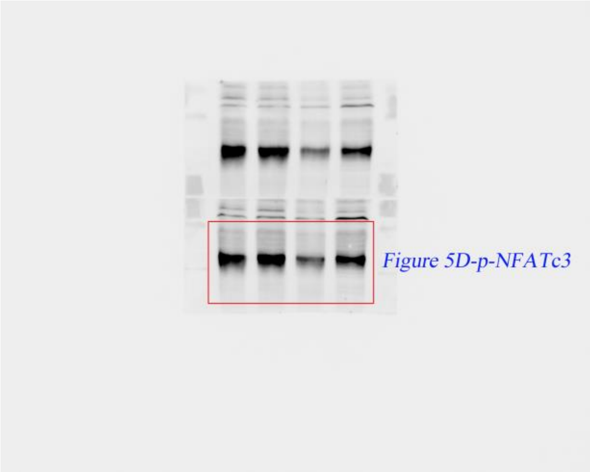

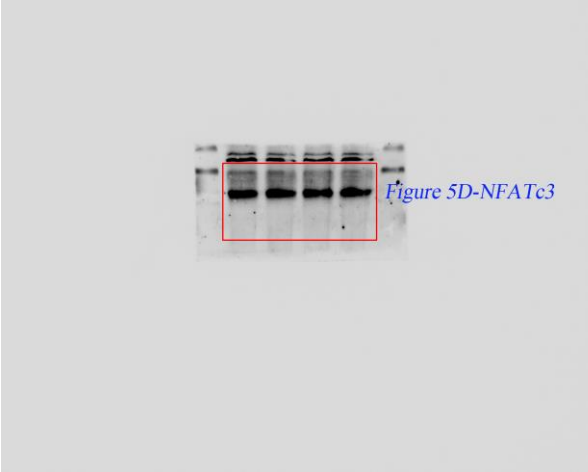

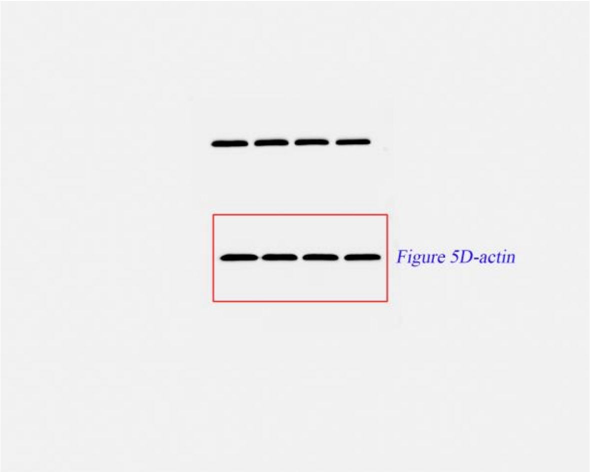

Figure 5F

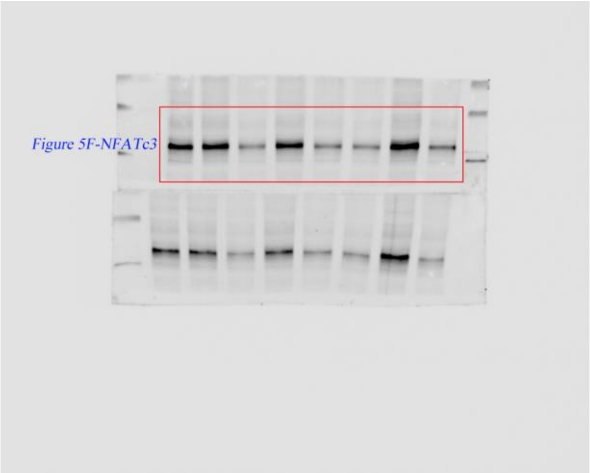

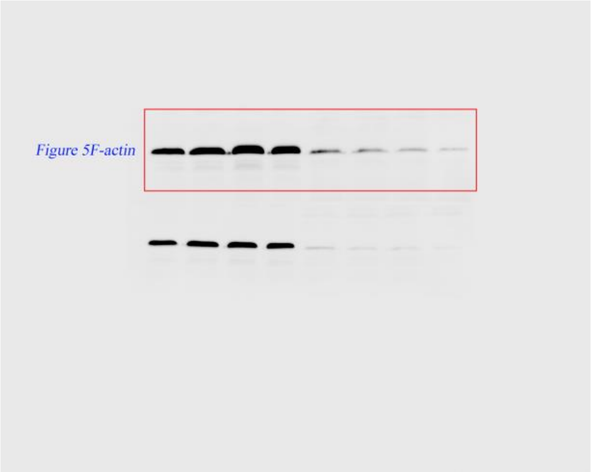

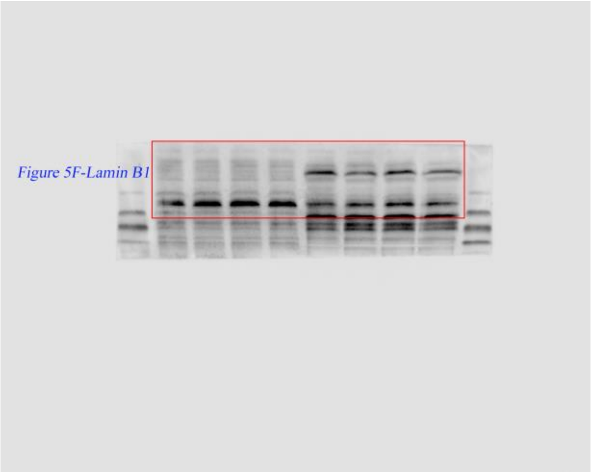
Figure 5F-Lamin B1
